# The trade-off between morphology and control in the co-optimized design of robots

**DOI:** 10.1371/journal.pone.0186107

**Published:** 2017-10-12

**Authors:** Andre Rosendo, Marco von Atzigen, Fumiya Iida

**Affiliations:** 1 Department of Engineering, The University of Cambridge, Cambridge, United Kingdom; 2 School of Information Science and Technology, ShanghaiTech University, Shanghai, China; 3 Institute of Robotics and Intelligent Systems, ETH Zurich, Zurich, Switzerland; University of Vermont, UNITED STATES

## Abstract

Conventionally, robot morphologies are developed through simulations and calculations, and different control methods are applied afterwards. Assuming that simulations and predictions are simplified representations of our reality, how sure can roboticists be that the chosen morphology is the most adequate for the possible control choices in the real-world? Here we study the influence of the design parameters in the creation of a robot with a Bayesian morphology-control (MC) co-optimization process. A robot autonomously creates child robots from a set of possible design parameters and uses Bayesian Optimization (BO) to infer the best locomotion behavior from real world experiments. Then, we systematically change from an MC co-optimization to a control-only (C) optimization, which better represents the traditional way that robots are developed, to explore the trade-off between these two methods. We show that although C processes can greatly improve the behavior of poor morphologies, such agents are still outperformed by MC co-optimization results with as few as 25 iterations. Our findings, on one hand, suggest that BO should be used in the design process of robots for both morphological and control parameters to reach optimal performance, and on the other hand, point to the downfall of current design methods in face of new search techniques.

## Introduction

As robots are, in their final form, physical representations of the model they were designed to be, discrepancies between design tools and reality augur poorly on their final behavior. This difference, known as the Reality Gap [1,2], can be reduced with programming methods to improve control parameters within the robot, but morphological parameters are difficult to modify and hence rarely changed, which incurs in a sub-optimal behavior.

Our central claim in this paper is that robots should be designed with a physical interplay between morphological and control parameters, which goes against the current trend of simulated designs being built on the basis of their best simulated behavior. Simulations are, to different degrees, simplifications of our physical world, and the lack of a tool capable of faithfully reproducing this real-world makes reality its best simulator. We present a design process where a robot creates another robot by choosing MC design parameters while inferring its real-world behavior. This robot uses BO to predict the influence of the chosen morphological and control parameters on the behavior of its child, and iteratively changes both parameters to find the best MC pair. Unlike current robotic applications with machine learning, where the control parameters are the only ones to be optimized in the real-world [3–5], this work introduces the idea of machines intelligently and purposefully designing their body and mind to reach a better real world behavior.

The idea of morphology and control jointly evolving is not new, and has been explored in simulated environments 20 years ago [6]. It has been approached over the years from different perspectives, such as optimizing control while fixing morphologies [7], using one simple control while optimizing morphologies around it [8], or even with a few simulations combining both traits, as seen in the implementations of neural networks combined with multi-legged systems [9]. The field advanced as an *in silico* analysis of evolution theories [10], and with Evolutionary algorithms (EAs) as the method of choice to simulate life. EAs are used in applications that transcend the virtual world [11], such as the design of an antenna [12] or robotic lifeforms [13], and it relies on random mutations and crossovers of elite genomes to reach a better performance. As these works are simulated until the last iteration, where the final output is built and tested, the efficacy of the solution will always depend on how well the Reality Gap was addressed. For many years, roboticists have focused on a higher understanding of control parameters, and these have been regarded as the basis for intelligent machines [14]. In this sense, the use of simulations as a prediction of morphological behavior was considered sufficient, and a higher-level control architecture was deemed enough to overcome morphological changes that a robot might suffer [15] or even to predict natural laws that govern their body [16]. This trend of prioritizing mind over body was eventually modified by works demonstrating passive-walking robots with highly stable gaits [17], and a new push for better morphological designs flourished with embodiment [18], self-reproducing robots [19] and soft robots [20].

It is undeniable that real-world experiments are needed to ensure the best morphological choice, but robotic morphologies are not easily changeable and design processes with a high number of iterations can slow the process drastically [21]. In a real-world experiment with evolutionary robots [22] a significant improvement in the final MC design could be seen, but the process required the construction of 500 robots to reach the best design. EAs are capable of optimizing systems, but the lack of an assumption over the influence of its parameters and the need to build every candidate makes it a poor method for MC optimization of robots. BO, on the other hand, establishes a relationship between M and C parameters to continuously update its expectations of behaviors [23]. This method has been used to design C parameters of a two-legged robot [2], to adjust C parameters to better adapt to morphological changes that the environment may apply against the robot [3], and to control robots and manipulators [24,25]. But how important are morphological considerations in initial steps of design? Additionally, can highly optimized C parameters on non-optimal morphologies eventually outperform an MC optimized agent?

## Results

Our experiments adopt the robot described in [21], which is a robotic manipulator with a gripper and a glue gun at the end effector. This robot, known as Mother Robot, autonomously assembles children robots by exploring two morphological and three control parameters, which results in a search space of 101,871 combinations. Each child is tested 50 times, and their fitness value estimated from the Euclidian distance from each trial to a target position.

**Fig 1:**
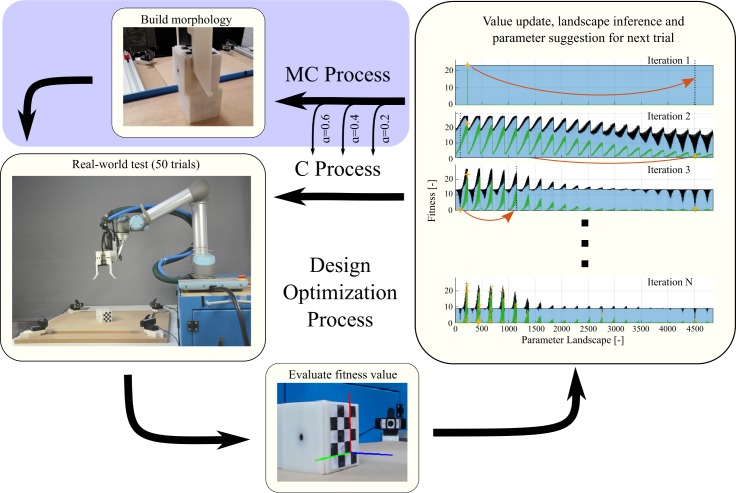
Overall system configuration. Mother Robot uses a gripper to rotate/move boxes, and glues them together. Within the test area the robot is systematically placed in the origin position, where control signals are applied. The outcome of each trial is recorded and evaluated, and the fitness value is used as an input to the BO, which infers the contribution from individual parameters to the fitness and suggests the next morphology to be built. In traditional engineering designs, tools such as CAE and physical simulators are used to approximate real-world behavior. Here, the design is autonomous and based on real-world data, without model approximations.

As we demonstrate in Fig 1, the system is capable of associating behavioral patterns from individual iterations to their respective outcomes, and uses BO to make this prediction. As a combination of Gaussian Processes [26] with Bayes theory, this method uses the target proximity from each iteration to predict, with an error margin, the behavior of unseen cases with similar traits, and the final output is an estimate of the entire parameter landscape. As the process progresses, Mother Robot alternates between exploratory iterations, to better understand other landscape options, and exploitative iterations, where the system focuses on the parameters in the neighborhood of the currently best ones.

### MC experiments

During our experiments we assigned time estimations to MC and C processes, as Mother Robot takes 15 minutes to create a new morphology and 30 minutes to perform and analyze trials. In this sense, performing an MC optimization is more time costly than a C optimization. As each iteration comprises 50 trials and we initially perform 50 MC iterations, it takes 37.5 hours to create these 50 morphologies and analyze 2500 trials.

**Fig 2:**
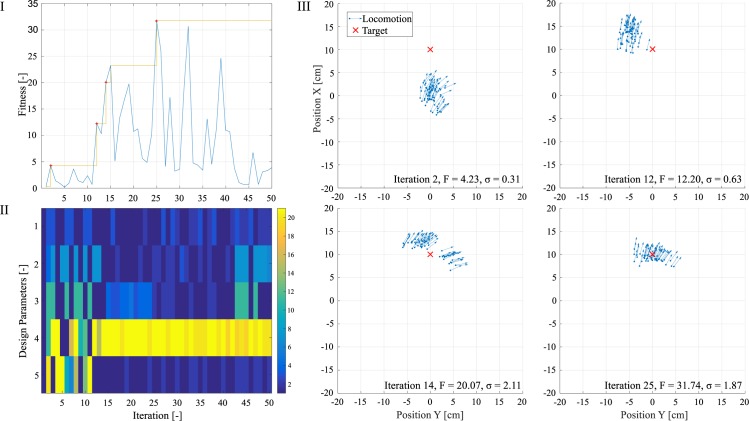
Morphology and control co-optimization. (I) The initial results of our MC co-optimization shows that the system gradually improves its performance until an optimum is reached. (II) The interplay between design parameters shows a highly exploratory initial behavior until iteration 12, followed by exploitative measures to discover the global optimum, and later returning to exploration to assure that the optimum is not local. (III) The trajectories for iterations 2, 12, 14 and 25 are highlighted with the end position and rotation of each trial, and their proximity to the target defines their fitness value.

The results of the MC experiments are depicted in Fig 2, and iteration number 25 represents the best design from the Mother Robot, where iterations 2, 12 and 14 show strong improvements over previous iterations. The locomotion seen in the first iterations barely leaves the start point, but the system approaches the target position as it acquires a better understanding of the influence of its non-trivial morphology to the environment. The design parameters were initially explored to later (iteration 12) be exploited by Mother Robot, which spends a long time in this mode (until iteration 42) to return to exploration.

### MC/C experiments

**Fig 3:**
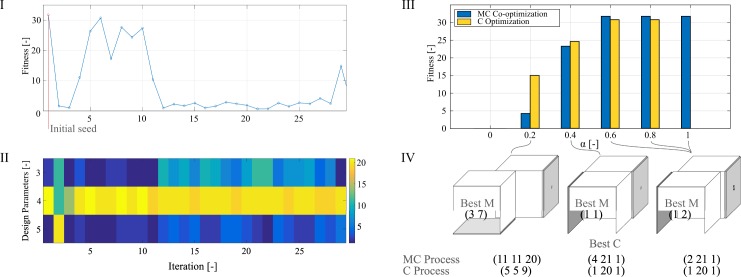
Bootstrapping C processes from MC. (I) The C optimization uses the best MC case as initial seed and tries to further improve it. (II) As observed with the MC optimization, the design parameters are initially explored to be later exploited until no further improvement is seen, eventually returning to landscape exploration. (III) The behavior of non-optimal morphologies, observed at ɑ = 0.2 and ɑ = 0.4, are further improved with the C optimization, while the last morphology found at ɑ = 0.6 and afterwards could not have its behavior further improved by C methods. (IV) The best control parameters observed at the end of MC and C processes reveal that small morphological changes, as observed between (1 1) and (1 2), can strongly influence the behavior, even if the control remains unchanged, i.e. (1 20 1).

How can Mother Robot know that investing its time in MC co-optimization is the best option? Current engineering practice finds a putatively optimal morphology and builds it so that engineers can fine-tune their control. In this vein, our secondary set of experiments aimed to interrupt the MC co-optimization in different iterative steps and to resume a C optimization on the nearly-optimal morphology, a strategy which we refer to as MC/C. We define the constant ɑ as the ratio between the amount of time spent on MC and the total experiment time of 37.5 hours. This new set of experiments is depicted in Fig 3.

Our experiments show that when 40% (ɑ = 0.4) or less of the design time was spent on an MC co-optimization the low performance from a bad morphology could be further improved with a C optimization, which bodes well to current engineering practices. However, when more than 60% of the initial design time is spent on an MC co-optimization the behavior of the system is superior to any other performance, and subsequent C optimizations do not improve the system further.

The strong influence of a good morphology can be seen at Fig 3 (IV), as control parameters converged to similar values, such as (1 20 1), but performances varied drastically with small morphological changes. The disparity between the performance shown between (1 1) and (1 2) is larger than those seen between the control parameters (2 21 1) and (1 20 1).

**Fig 4:**
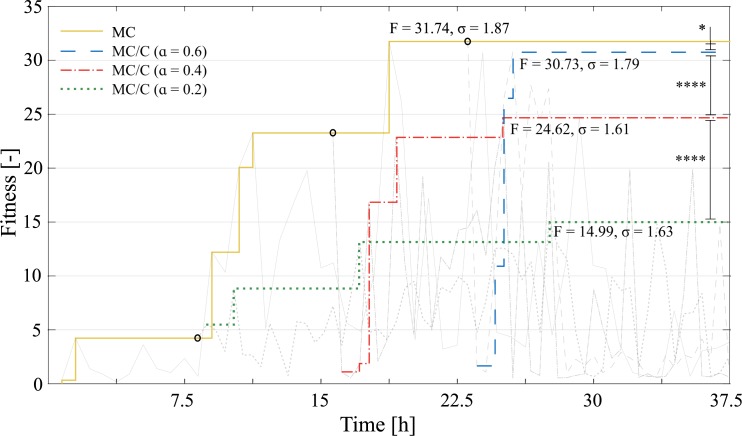
Superiority of MC over MC/C. The maximum fitness found after 37.5 hours of MC experiments is shown with a yellow line. The C processes of ɑ = 0.2 (green), ɑ = 0.4 (red) and ɑ = 0.6 (blue) are shown with their initial seed depicted with a black circle. A post-hoc Tukey HSD test performed in the best result from each process shows statistical significance (****, p<0.001, or *, p<0.05) for all cases. The importance of a good morphology is highlighted by the improvements observed from green to red and from red to blue. Still, the green, blue and red lines are always below the values of the golden line. Thus, the MC process is faster than the three C alternatives in finding better solutions.

Although results from Fig 3 (III) apparently suggest that C optimizations were better than MC until ɑ = 0.4, this comparison is not a fair one as the design time is not taken into consideration. When each design alternative is analyzed from a time perspective, as done in Fig 4, it is clear that the results obtained by the MC process are superior to the results from MC/C processes. The time to generate a superior performance with C processes is longer than an MC process would take to find a better solution.

## Discussion

The quality of the morphology plays a crucial role in robotics, and physical experiments are required to make sure that the interplay between morphology and control is as intended during real-world performance. In the past, simulations were combined with experiments to bootstrap designs [13], but no guarantee could be made on the real-world behavior, and a stark behavioral difference between simulated and experimental results was observed in their results. This Reality Gap [1] introduces discrepancies between modeled and real environments, and amplifies the divergence between final performances. Our work differs from these previous model-driven results by using a data-driven approach, and although our experiments lack a model, these results show that it is possible to reach the best real-world performance without one and with a limited number of iterations. Simulations combined with experiments carry over simplifications and, as a result, behavioral differences emerge [1,2,13].

### Design with Bayesian Optimization

Bypassing the Reality Gap with a fully experimental design seems like a sensible solution, but the idea is hindered by the high number of iterations to reach the final design. As designers, we imagine how things are supposed to be or behave, and act upon this “mental model” to fulfill our biases. Optimization methods, on the other hand, usually do not have biases and solely rely on assumptions concerning the observed data. As a direct example, [21] demonstrates the construction of robots with EA in a model-free approach. Our work contrasts with the EA approach by introducing BO into the MC process. The lack of an “intelligent” mechanism within EA deems the real-world optimization prohibitively costly, and requiring the construction of 500 robots. Our work strategically searched the design landscape and the global optimum was achieved within 25 iterations.

This fairly small number of iterations is possible with the use of BO to allow inferences over the behavior of design parameters which were still not tested. Consequently, the chances of restraining the optimization process to a local maximum decrease with iterations. As a better notion of the entire design landscape is possible, as exemplified in Fig 1, our process “intelligently” samples parameters which have higher potential to reduce uncertainties in our predictions. In Fig 2 we can see the concrete benefit of this approach in robotics, and although the same BO has been solely used to tune C parameters in legged robots [3,4], we show for the first time the benefits of MC co-optimization.

### Morphology and control trade-off

As the MC co-optimization process advances, the system suffers gradually smaller morphological shifts, converging from the morphology (1 1) at ɑ = 0.4 to the morphology (1 2) at ɑ = 0.6. The optimality of each morphology can be explained by the comparison of maximum fitnesses from Fig 3, with some gradual C improvements seen with morphologies (3 7) and (1 1), and eventually the optimal control found by the MC process within (1 2).

Although the idea of bootstrapped complex behaviors is present in previous works [16], in our experiments the bootstrapped C optimization (ɑ = 0.2, 0.4 and 0.6 from Fig 4) is almost always worse than an uninterrupted MC optimization. This prompts us to rethink how engineering designs are conceived, as morphological traits are usually locked in the very first stages of development. Design methods, such as CAD and FEM, are crucial for morphological predictions, but they are usually disconnected from physical simulations, and thus C happens after M. While an opposite approach would be to optimize an M parameter for a specific C parameter [12], in our previous work [21] we introduced a nested development stage to an EA, where some M parameters evolved while C parameters were frozen, and the improvement of the system depended heavily on which M parameter was chosen to evolve. In here, we show for the first time an analysis of the trade-off between morphology and control of robots in real-life.

In a parallel between our work and a walking robot [15], where the robot creates an internal model (M) while interacting (C) with the environment, the results from both works strongly agree over the superiority of the process when M and C take place concomitantly, as opposed to separating these two processes. In this sense, and observing the results from Fig 4, design time should never be spared when co-optimizing morphology and control, as this method is superior to a control optimization of a constrained morphology.

### Study limitations

As this is the first time that BO co-optimizes M and C and that a parallel between this method and a C-only optimization is made, our results are strongly influenced by the adopted morphology and the size of the search space. We tried to create a wide search space to mimic a diversified design condition, and an even wider morphological search space, with three or four cubes assembled together, would make the search for the best morphology more difficult and, consequently, would increase the chances of a C-only process to fall into a local maximum (i.e. control optimization of a sub-optimal morphology).

A wider search space of C parameters would have required a more detailed search for different ɑ values, and different building times or experimenting times would also require more trials. Future works could complement this work with the addition of different morphologies, and parallels between their complexities could enhance our understanding of how these influence the design of robots.

## Conclusion

In this work we discussed the influence of morphological and control parameters on the design of robots with an autonomous experimental setting. Although the co-optimization of morphology and control requires a parametric search within a wider search space, this MC search yielded better and faster results than a control-only search. This result points to the downfall of robotic design methods when morphologies are constrained in the initial development stage while control is given priority.

This work also uses BO to design robots, going one step further from previous works (3,4), which solely used this optimization technique to adapt the control method to a morphology. BO is capable of inferring the behavior of unknown parameters from parameters which were already tested, and this ability reduces the need for a higher number of iterations. A formal comparison between BO and EA was not available, but the speed at which the system converged to the global optimum leads the authors to believe that BO is better in solving MC design problems.

Soft robotics enables a safer interaction between humans and robots, but introduces morphological uncertainties to locomotion and manipulation problems. If robots could actively change their morphology and control to better perform a task the aforementioned shortcoming would become a much desirable ability, and MC co-optimization would be essential in this scenario.

## Materials and methods

### Mother and child robots

The robot referred to as Mother Robot throughout this article is a UR5 manipulator from Universal Robots, and its role is to assemble smaller modular robots which we call “children”, and both robots are shown in Fig 1. The end effector of UR5 has both a pneumatically actuated gripper and a mounted glue valve. The hot glue is supplied by a glue tank and a heated hose. Mother Robot can either manufacture child robots by gripping active modular cubes and assembling them with hot glue, or Mother Robot can perform pick and place actions with child robots.

The child robot consists of two modular active cubes surrounded by an Acrylonitrile butadiene styrene (ABS) shell and with a side length of 6 cm. Each cube comprises of a battery, an Arduino, a Bluetooth module and a servo motor attached to a rotating face.

### Morphological and control parameters

The creation of machines by machines is difficult due to the almost infinite amount of options to assemble two cubes. As a consequence, there is a need to discretize both morphological and control parameters to enforce a finite number of combinations.

**Fig 5:**
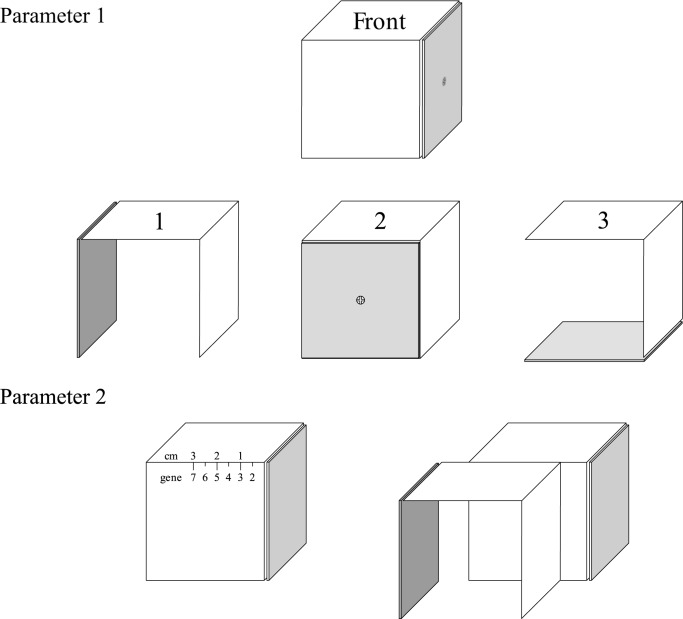
Morphological parameters. The morphological parameters of the child robot can be seen above. While the first parameter determines the orientation of the second cube in respect to the first, the second parameter determines the off-set between these cubes. In the example above, the first parameter is 1 while the second parameter is 3, with a 1 cm gap between the cubes.

The morphology is defined by two parameters. The first parameter describes the orientation of the rear cube with respect to the front cube where the orientation of the front cube is fixed. The second parameter allows the two cubes to be offset by a certain distance, and the value of this parameter ranges from one to seven and covers a displacement between 0cm and 3cm, in 0.5cm steps. The distance is always measured perpendicularly from the edge that is nearest to the rotating face. Both parameters are depicted in Fig 5.

**Fig 6:**
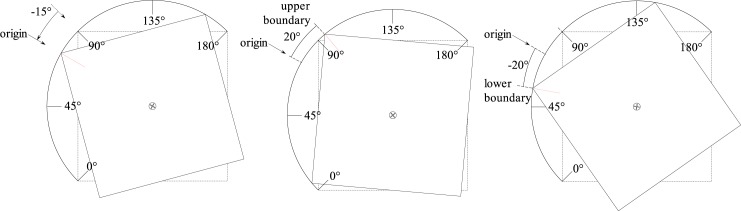
Angular amplitude of the cube. The amplitude, the third parameter from the child robot, determines the deflection from the origin position in either direction. The front and rear cubes can have different values for their origins, defined as parameters 4 and 5, respectively. In the figure, the origin is 90°-15° = 75°, while the amplitude is 20°.

The control parameters are more trivial to discretize. While amplitude corresponds to the maximum deflection in degrees from the zero position of the rotation face with respect to the cube, the aforementioned zero position of the rotating face with respect to the cube can be set to different values to create an angular offset between cubes. Preliminary experiments indicated that a change of two degrees in either the amplitude of movement or the origin of rotation affects the behavior noticeably, and thus the control parameter values for these is sampled in increments of two degrees. Fig 6 depicts the influence of amplitude (parameter 3) and origins (parameters 4 and 5) on the behavior of the cube.

### Experimental method

Our experiments with Mother Robot consisted of creating a child robot with a specific morphology and control input, defined by the aforementioned parameters, placing this child on the testing platform and performing 50 trials with the same MC parameters (each of these trials lasted 2500 milliseconds). The need for such high repeatability comes from the stochastic noise that real-world experiments present, and we used a fully automated system to overcome this problem. The mean value and standard deviation of these 50 trials were used for a one-way ANOVA test with a Tukey HSD post-hoc test considering a 95% confidence interval.

The automated system stores information from the pose estimation system to calculate the position of the child robot and to evaluate the fitness of the robot, and Bayesian Optimization (BO) uses this information to infer the next parameter set to try.

### Pose estimation

Although the system consists of two robots, the Mother Robot has its base in a static platform and this facilitates the mathematical definition of the end-effector in relation to the testing platform. The child robot, on the other hand, moves freely within the testing platform, and the 3D pose estimation is crucial to record the final position of this robot and to pick and place it after each trial is done.

The pose estimation comprises 4 cameras, one algorithm to recognize the chessboard pattern and one to algorithm to place the extracted pattern within one global reference frame. In our previous works we opted for a background subtraction and a feature extraction to identify robots [22], but this method required a high computation power in parallel to the homeostasis of our system (Trajectory of the end-effector, BO calculations, etc.). In here we opted for a chessboard pattern extraction, commonly available as a library for OpenCV, in which the system simultaneously identifies the corners of a 4x5 chessboard in four cameras.

After locating the chessboard pattern an algorithm calculates the rotation and translation of the vector and transforms the coordinates of the visible patterns into one single reference frame. The system runs in a multithreaded computer at a rate of 20 fps, which is fast enough since our experiments are only interested in recording the initial and final positions.

### Bayesian optimization

BO can be seen as an extension to Gaussian Processes, which are used to map input-output relationships of processes together with an uncertainty of the mapping [26]. Gaussian Processes propagate a mean estimate μ and a variance estimate σ^2^ for every point in the input space and they adapt to new data by changing their hyperparameters automatically to conform to all available information. Different flavors of this same process can be found in different robotic applications, such as sensing and trajectory [27, 28].

Given a set of observations **f**_1:t_ = f(x_1:t_) of the fitness function at the data points **x**_1:t_, the Gaussian Process is given as N(0,**K**) where **K** denotes the covariance matrix with measurement noise added to the diagonal entries.

$$K=\left[\begin{array}{ccc} k(x_{1},x_{1}) & \cdots & k(x_{1},x_{t})\\ \vdots & \ddots & \vdots \\ k(x_{t},x_{1}) & \cdots & k(x_{t},x_{t}) \end{array}\right]+\sigma_{meas}^{2}I$$

With a covariance kernel k. Popular covariance kernels are the Squared Exponential (SE), the Matérn or periodic kernels. The most flexible kernel from this selection is the Matérn kernel which is able to map both smooth regions as well as sharp edges. The periodic kernel, even though promising for these experiments, was not chosen due to the implicitly imposed periodicity that counteracts our efforts to leave the algorithm unbiased, flexible and adaptable. The Matérn covariance kernel is represented by

$$k_{Matern}(d)=\sigma_{p}^{2}\frac{2^{1-\nu }}{\Gamma (\nu )}{\frac{\sqrt{2\nu d}}{l}}^{\nu }K_{\nu }\frac{\sqrt{2\nu d}}{l}$$

where ν is a positive parameter, Γ denotes the gamma function and Kν represents a Bessel function. The process noise variance is denoted by σp2 and l represents the characteristic length-scale. This function reduces to the square exponential as ν approaches ∞. The Matérn kernel simplifies for half integer values of ν. Choosing ν = 5/2 results in the following covariance function:

$$k_{\nu =5/2}(d)=\sigma_{p}^{2}\left(1+\frac{\sqrt{5}d}{l}+\frac{5d^{2}}{3l^{2}}\right)e^{-\frac{\sqrt{5}d}{l}}$$

At time t+1 a new observation f_t+1_ at location x_t+1_ is available and has to be incorporated into the Gaussian Process.

$$\left[\begin{array}{c} f_{1:t}\\ f_{t+1} \end{array}\right]∼N\left(0,[\begin{array}{cc} K & k\\ k^{T} & k(x_{t+1},x_{t+1}) \end{array}]\right)$$

Where the bold **k** is:

$$k=[k(x_{t+1},x_{1})\;k(x_{t+1},x_{2})\;\ldots \;k(x_{t+1},x_{t})]$$

This results in the two update equations for the mean μ and the variance σ^2^.

$$\mu_{t}(x_{t+1})=k^{T}K^{-1}f_{1:t}$$

$$\sigma_{t}^{2}(x_{t+1})=k(x_{t+1},x_{t+1})-k^{T}K^{-1}k$$

With this input-output mapping at hand, BO selects the next query point by maximizing an acquisition function. Again, there are multiple valid choices, e.g. the Probability of Improvement (PI) function describes the probability of finding a fitness feedback f(x) at data point x that exceeds the currently best fitness feedback f(x^+^) by ξ. It is worth noting that a high value for ξ will promote exploration, since region will be investigated, where the uncertainty is high, imposing a high potential to improve the currently best fitness. Accordingly, a small ξ will promote exploitation, since high performance regions are more likely to improve the solution slightly:

$$PI(x)=P(f(x)\geq f(x^{+})+\xi )$$

$$PI(x)=\Phi \left(\frac{\mu (x)-f(x^{+})-\xi }{\sigma (x)}\right)$$

Where Φ denotes the normal cumulative distribution function. Another approach is to minimize the expected deviation from the true maximum f(x*), which evaluates to the following:

$$EI(x)=\left\{ \begin{array}{l} (\mu (x)-f(x^{+})-\xi )\Phi (Z)+\sigma (x)\phi (Z)\;\mathrm{if}\;\sigma (x)>0\\ 0\;\mathrm{otherwise} \end{array}\right.$$

$$Z=\frac{\mu (x)-f(x^{+})-\xi }{\sigma (x)}\;\mathrm{if}\;\sigma (x)>0$$

Where Φ again stands for the normal cumulative distribution function and *ϕ* denotes the normal probability distribution function.

This interplay between updating the mapping based on new data and suggesting the next data point is key to BO. Compared to other optimization algorithms, BO estimates a fitness function value for every possible data point x by inferring the performance of unseen data points in a probabilistic fashion.

### Data availability

The results of this experiment, with each of the 50 trials per iteration for the MC cases (ɑ = 1) and for the bootstrapped C cases (ɑ = 0.2, ɑ = 0.4 and ɑ = 0.6) can be found at the Open Science Framework at the following link: https://osf.io/fnh7v/download

## Supporting information

S1 Movie Autonomous MC tests. In our experiments the trade-off between Morphology and Control are examined, and a control-only methodology is bootstrapped from different steps of the MC optimization.
(MP4)
